# Cold spell *en route* delays spring arrival and decreases apparent survival in a long-distance migratory songbird

**DOI:** 10.1186/s12898-017-0121-4

**Published:** 2017-04-04

**Authors:** Martins Briedis, Steffen Hahn, Peter Adamík

**Affiliations:** 1 Department of Zoology, Palacký University, tř. 17. listopadu 50, 77146 Olomouc, Czech Republic; 2 Department of Bird Migration, Swiss Ornithological Institute, Seerose 1, 6204 Sempach, Switzerland; 3 Museum of Natural History, nám. Republiky 5, 77173 Olomouc, Czech Republic

**Keywords:** Circannual rhythm, Climate change, Geolocator, Long-distance migrant, Phenology, Weather extremes

## Abstract

**Background:**

Adjusting the timing of annual events to gradual changes in environmental conditions is necessary for population viability. However, adaptations to weather extremes are poorly documented in migratory species. Due to their vast seasonal movements, long-distance migrants face unique challenges in responding to changes as they rely on an endogenous circannual rhythm to cue the timing of their migration. Furthermore, the exact mechanisms that explain how environmental factors shape the migration schedules of long-distance migrants are often unknown.

**Results:**

Here we show that long-distance migrating semi-collared flycatchers *Ficedula semitorquata* delayed the last phase of their spring migration and the population suffered low return rates to breeding sites while enduring a severe cold spell *en route*. We found that the onset of spring migration in Africa and the timing of Sahara crossing were consistent between early and late springs while the arrival at the breeding site depended on spring phenology at stopover areas in each particular year.

**Conclusion:**

Understanding how environmental stimuli and endogenous circannual rhythms interact can improve predictions of the consequences of climate changes on migratory animals.

**Electronic supplementary material:**

The online version of this article (doi:10.1186/s12898-017-0121-4) contains supplementary material, which is available to authorized users.

## Background

Over the course of the 20th century, the Earth’s near-surface temperature has increased, [1] and many species have advanced their phenology as a response to this climate warming [2]. Among those, various migratory birds have advanced their spring migration and breeding schedules [3], with stronger responses in short-distance compared to long-distance migrants [4].

Long-distance migrants spend the non-breeding period in the areas where they often have limited possibilities to assess the climatic conditions at their distant breeding grounds, thus limiting their ability to time the spring migration accordingly. Current theory suggests that long-distance migratory birds depend on endogenously controlled circannual rhythms to cue their spring migration [5, 6]. Photoperiod and environmental factors may serve as *Zeitgeber* to fine-tune the timing of departure [7–9]. While the mechanisms regulating the onset of spring migration are not yet fully understood, even less is known about the processes modifying migration rates and decision making *en route* [10]. Thus, the specific factors that determine the observed advances in spring arrival of long-distance migrants remain unknown.

The understanding how animals respond to the changing environment is of special importance with respect to increasing frequency of extreme weather events [11]. Inability to respond to a rapidly changing environment can have severe consequences on population demography and viability. If long-distance migrants rely solely on endogenous signals to time the entire spring migration, this could result in suboptimal arrivals at the breeding sites, possibly leading to mismatches of food peak availability and food demand [12].

**Fig. 1:**
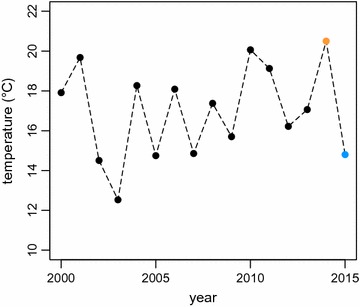
Average land surface temperature in Bulgaria, Greece and Turkey from 22 March to 7 April from 2000 to 2015 [Data available from the U.S. Geological Survey (http://www.usgs.gov/)]

Here we examine how long-distance migrating semi-collared flycatchers *Ficedula semitorquata* respond to contrasting climatological conditions encountered in two consecutive spring migrations. Flycatchers’ peak arrival period at their breeding range extends from the end of March to the beginning of April [13]. In Southeastern Europe in 2014, this period was the warmest on record since 2000, followed by an exceptional cold spring in 2015 with temperatures well below the long-term average (Fig. 1). Such extreme and opposing conditions present an ideal opportunity to study phenotypic plasticity in a natural setting. We were particularly interested to test whether this obligatory long-distance migrant is capable of adjusting its migration rate based on environmental cues *en route* to fine-tune arrival at the breeding site.

## Methods

### Study site and geolocators

Our study site is located in eastern Bulgaria (42°55′N, 27°48′E) approximately 8 km from the Black Sea coast at 120–150 masl. Habitat at the breeding site is oak woodland dominated by Hungarian oak *Quercus frainetto* with very little undergrowth. A population of approximately 100 pairs of semi-collared flycatchers breeds in nest boxes.

During the breeding season of 2013 and 2014 we equipped 40 (17 males, 23 females) and 49 (27 males, 22 females) adults with geolocators (GDL2.0, Swiss Ornithological Institute; weight including the harness: 0.6 g) which were fitted on birds’ backs using elastic leg-loop silicone harnesses. The geolocators on average constituted 4.6 ± 0.3% (SD) of the bird’s body mass. There was no difference in the average load of the geolocator between the birds that returned and those that did not return (average ± SD; returned: 4.7 ± 0.3%, n = 18; not returned: 4.6 ± 0.3%, n = 71; t test: t_87_ = −1.19, p = 0.25).

We did an extensive recapturing of the tagged birds upon their arrival at the breeding site. Birds were captured using mist-nets and traps inside the nest boxes before the initiation of nest building. All adult breeders were captured later in the season when feeding nestlings, allowing for additional geolocator retrieval from the birds not captured earlier. In total we recovered 18 geolocators (2014: n = 11, 2015: n = 7); however, due to technical problems, we obtained spring migration data from only 5 [2 females, 3 males (1 incomplete)] and 6 (2 females, 4 males) devices in 2014 and 2015, respectively.

In addition, we acquired spring migration passage dates of flycatchers from the Antikythira Bird Observatory, Greece (35°51′N, 23°18′E, [14]) from 2007 to 2015.

### Data analysis

We processed the light recording data using the R-package ‘GeoLight’ v2.0 [15], having determined sunrise and sunset times with ‘Geolocator’ software (Swiss Ornithological Institute) beforehand. We filtered the datasets for outlaying sun events using the ‘loessFilter’ function (k value = 2). We determined departure from the non-breeding site and arrival at the breeding site using the ‘changeLight’ function (probability of change q = 0.8). Minimum stationary period duration was set to 3 days. We determined Sahara crossing time according to the procedure described by Adamík et al. [16]. In short, during the Sahara crossing days geolocator’s light sensor records uninterrupted maximal light intensities throughout the day, suggesting that birds cross the ecological barrier with a non-stop flight or at least prolonging the typical nocturnal flight for several hours into the following day. We adjusted the probability of change in the ‘changeLight’ function for each individual starting from q = 0.8, so that the function detects Sahara crossing time as a movement period. Annual timing of key migration phases are given as median date plus interquartile range (IQR) throughout.

To test for differences in apparent local survival rates between 2013–2014 and 2014–2015, we used a Chi squared goodness-of-fit test without Yates correction.

### Weather data acquisition

We obtained land surface temperature data (data set: MOD11A2) and leaf area index (MOD15A2) data during the spring migration period (10 February–7 April) from MODIS terra and aqua satellites, accessed from the Land Processes Distributed Active Archive Center (LP DAAC) at the US Geological Survey (USGS) Earth Resources Observation and Science (EROS) Center (https://lpdaac.usgs.gov/). We obtained wind data for the 850 mb pressure level (approximately 1500 masl) from the National Center for Environmental Prediction (NCEP)/National Center for Atmospheric Research (NCAR) Reanalysis dataset [17] using R-package ‘RNCEP’ [18]. Data were gathered across a 2.5° grid for every 6 h period in 2014, 2015 and annually averaged across the whole spring migration period (10 February–7 April). Winds at the 850 m bar pressure level are largely free of orographic distortion and, thus, are frequently used for describing wind patterns experienced by migratory birds [19].

## Results

### Weather patterns

**Fig. 2:**
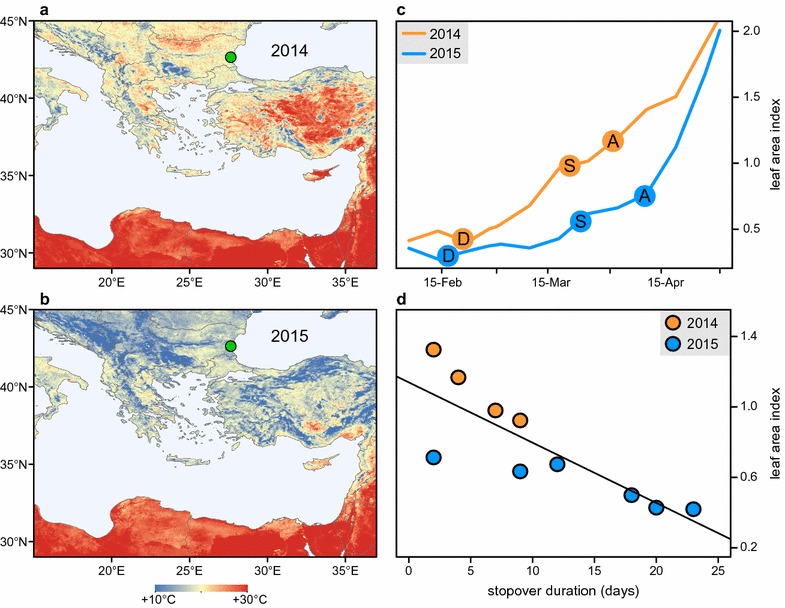
Annual differences in weather conditions and the corresponding migration phenology of semi-collared flycatchers. Land surface temperatures (°C) from 22 March–7 April in **a** 2014 and **b** 2015. **c** Leaf area index (m^2^ of leaf area per m^2^ ground area) progression from 6 February–1 May at the flycatcher’s breeding site in 2014 (*orange*) and 2015 (*blue*) and the related flycatcher migration phenology in each year, including (D) departure from the non-breeding site, (S) Sahara crossing, and (A) arrival at the breeding site. **d** Stopover duration north of the Sahara in relation to leaf area index at the breeding site at the time of Sahara crossing [The background maps in **a** and **b** made were made from data available from the U.S. Geological Survey (http://www.usgs.gov/); maps were created in ArcMap 10.1 (http://www.esri.com/)]

**Fig. 3:**
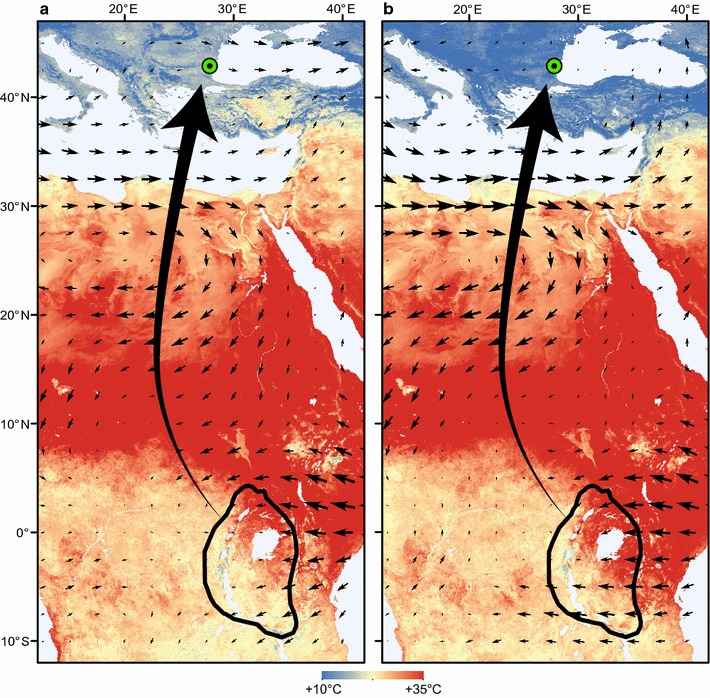
Average land surface temperature (*background map*) and wind patterns at 850 kPa pressure level (*small arrows*) during semi-collared flycatcher spring migration from 10 February to 7 April in **a** 2014 and **b** 2015. *Black shape* outlines semi-collared flycatcher non-breeding range (BirdLife International and NatureServe 2011) and *large arrows* indicate spring migration routes [Temperature data available from the U.S. Geological Survey (http://www.usgs.gov/); wind data available from National Oceanic and Atmospheric Administration (http://www.noaa.gov/). Maps were created in ArcMap 10.1 (http://www.esri.com/)]

The average land surface temperature during the spring migration period across Bulgaria, Greece and Turkey—countries on the species flyway—from 22 March–7 April was 20.5 °C in 2014, while in 2015 it was only 14.8 °C (Fig. 2a, b). This was the largest such difference in air temperature for over a decade (Fig. 1). Plant phenology, measured by leaf development, was delayed by approximately 29 days in 2015 compared to 2014 (Fig. 2c). Along other parts of the flycatchers’ migratory flyway of the flycatchers, the prevailing winds and temperatures were similar between the two study years (Fig. 3).

### Responses of migrants

During both years, flycatchers departed from their non-breeding grounds in Eastern-Central Africa in the second half of February {median date 2014: 21 February [interquartile range (IQR) = 17–22 Feb], 2015: 16 February (11–19 Feb), Fig. 2c} and crossed the Sahara desert in late March [2014: 23 March (17–30 Mar), 2015: 27 March (21 Mar–5 Apr)]. After crossing the Sahara, the birds stayed in the Mediterranean Basin for 5 days (3.5–7.1) in 2014 before arriving at the breeding site on 2 April (29 Mar–7 Apr). In 2015 birds spent three times longer (mean 15 days, IQR 9.8–19.5) in the Mediterranean Basin and arrived at the breeding site on 10 April (9–11 Apr, see Additional file 1). We found a negative relationship between the time spent in the Mediterranean Basin and leaf development at the breeding site (Pearson’s one-tailed correlation: r = −0.82, n = 10, p = 0.002; Fig. 2d). The median spring migration passage times of flycatchers at Antikythira Bird Observatory in 2014 and 2015 were within the species’ typical long-term passage period (2014: 17 Apr; 2015: 14 Apr; 2007–2015: 15 Apr, IQR = 12–18 Apr).

**Fig. 4:**
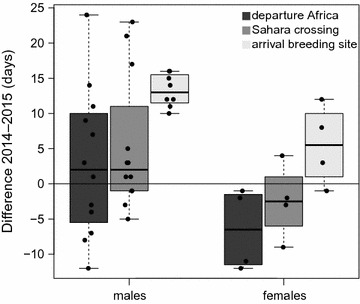
Sex-specific difference in individual migration schedules between a cold (2015) and a warm spring (2014). The delay in arrival is larger for the earlier migrating males than for later migrating females. Individual data points represent difference between every possible pair of two individuals tracked in different years

We also observed prominent sex differences in migration timing, with males crossing the Sahara and arriving at the breeding site earlier than females in both years. The distinct protandry resulted in stronger delays in males’ migration schedule than in females’. In 2015 males arrived at the breeding site on average 13 days later than in 2014, while the difference for females was only 5.5 days (Fig. 4).

The cold spell of 2015 also had severe consequences on apparent local survival. Return rates of geolocator-tagged and ringed-only control birds were approximately two times lower in 2015 (Table 1), with males and older individuals (more than 3 years old) affected more severely.

**Table 1:** Differences in return rates of semi-collared flycatchers between 2014 and 2015, and between control group and geolocator-tagged group

	2014	2015	χ^2^	p value
Control				
Males	58.8% (30/51)	23.8% (19/80)	6.29	*0.01*
Females	38.1% (24/63)	21.7% (15/69)	1.78	0.18
2cy	64.3% (18/28)	51.4% (19/37)	0.11	0.74
2cy+	41.9% (36/86)	13.4% (15/112)	10.90	*<0.001*
Total	47.4% (54/114)	22.8% (34/149)	7.87	*0.005*
Tagged				
Males	47.1% (8/17)	18.5% (5/27)	1.31	0.25
Females	13.0% (3/23)	9.1% (2/22)	1.4e−30	1
2cy	37.5% (6/16)	21.4% (3/14)	0.11	0.75
2cy+	20.8% (5/24)	11.4% (4/35)	0.23	0.63
Total	27.5% (11/40)	14.3% (7/49)	0.99	0.32

	Control	Tagged	χ^2^	p value
2014–2015				
Males	37.4% (49/131)	29.5% (13/44)	0.24	0.63
Females	29.5% (39/132)	11.1% (5/45)	3.22	0.07
2cy	56.9% (37/65)	30.0% (9/30)	1.68	0.20
2cy+	25.8% (51/198)	15.3% (9/59)	1.39	0.24
Total	33.5% (88/263)	20.2% (18/89)	2.69	0.10

Significant differences are given in italics

## Discussion

Our findings show that a cold spell encountered *en route* delayed spring arrival and decreased local apparent survival in a trans-Equatorial migrant. After reaching the temperate climatic zone where environmental cues of spring phenology become available, tracked semi-collared flycatchers flexibly adjusted their migration rate and advanced (in the warm spring of 2014) or delayed (in the cold spring of 2015) their arrival at the breeding site depending on local conditions (e.g. temperature and leaf development).

The typical passage times of semi-collared flycatchers at Antikythira Bird Observatory range from the end of March to the end of April [14], with most birds passing in the second decade of April. Median passage times in the second decade of April may imply that the flyway through Antikythira is used by different populations than ours, and those populations migrating through Antikythira do not pass there until after the prolonged stopovers of our tracked birds in 2015. Indeed in 2014, birds from our study population arrival at the breeding site earlier than the median passage time at Antikythira, supporting this idea.

So far, contrasting results have been reported in long-distance migrants regarding their ability to use environmental signals to cue spring migration [5]. Nearctic-Neotropical long-distance migrants have been shown to use environmental cues to some extent to adjust their migration rate in spring [20, 21]. On the contrary, pied flycatchers *Ficedula hypoleuca* were not able to adjust the arrival time proportionally to the increasing spring temperatures suggesting a tight endogenous routine controlling phenology of spring migration in that population [22]. Recent tracking studies confirm these findings, showing that breeding site arrival date in pied flycatchers largely depends on the onset of spring migration, rather than birds making adjustments *en route* [23]. In the closely related collared flycatcher *F. albicollis*, spring arrival at different breeding sites is related to local phenology, and timing of the onset of spring migration seems to be less important [24]. This coincides with our findings in semi-collared flycatchers. The differences between these three *Ficedula* species may be related to the migratory flyway they use during the spring migration. Resource availability and ecological barriers encountered *en route* can influence on the rate and timing of bird migration [25, 26]. Species that encounter ecological barriers along the migratory flyway and have larger migratory distance show a greater degree of variation in their migratory behaviour and ability to adjust migration rate in response to the environment. In spring, pied flycatchers migrate along the western Afro-Palearctic flyway, while collared and semi-collared flycatchers migrate along the central Afro-Palearctic flyway. Migrants using the central Afro-Palearctic flyway encounter larger ecological barriers (e.g. the distance to cross the Sahara Desert is larger) and harsher conditions compared to the species using the western Afro-Palearctic flyway.

To date there seems to be no general consensus on where along a migration route the changing conditions should have the largest effect on the timing of bird arrival [10]. Tøttrup et al. [27] demonstrated that drought in the Horn of Africa delayed spring arrival of Afro-Palearctic migrants, as birds prolonged their stopovers in this area. This, when considered with our results suggests that prolonged stopovers due to adverse weather conditions could occur at any place along the migratory route (in the tropics and temperate regions alike), and can cause delayed arrival at the breeding sites.

As a consequence of adverse weather, increased mortality rates have previously been reported across different taxa [28]. Our finding of low apparent survival of flycatchers in a year with adverse weather conditions likely indicates increased mortality. Alternatively, birds may have acted opportunistically and settled for breeding elsewhere along the migratory route or exhibited a higher degree of breeding dispersal compared to the previous year. In our study, males showed lower return rates than females in the colder spring of 2015. By arriving earlier, males are exposed to a more hostile environment, including lower food availability, than later arriving females. Similarly, older flycatchers usually arrive at the breeding site earlier than younger ones and would therefore undergo similar consequences to those of males versus females. In cliff swallows *Petrochelidon pyrrhonota* higher mortality of older individuals was found as a result of a cold spell, coinciding with our findings of low return rates [29].

Geolocator attachment has been shown to negatively affect return rates of birds [30]. However the recent evidence is ambiguous, with a number of studies showing no apparent effect on return rates of the tagged birds [e.g. 31, 32], while some report negative influence [33] including delayed breeding site arrival time and decreased breeding success in the year following the geolocator deployment [34]. Furthermore, the differences in return rates between tagged and control birds seem to vary among sites within the same species [24, 35]. Therefore, having a control group of ringed only individuals within a study population is recommended in order to evaluate the impact of the attached devices on the animals. It may be that the limited sample size of tagged birds restricted our ability to detect a significant negative effect on individual apparent survival associated with carrying the geolocator, despite the fact that return rates of the geolocator-tagged individuals in our study were lower than for ringed only birds (see Table 1). However, we have no reason to believe that the extra weight of the geolocators influenced the migration speed and stopover behaviour of our study birds, as our field observations show simultaneous arrival of the tagged and ringed-only birds.

## Conclusions

Our tracked flycatchers prolonged their stopovers in the Mediterranean region when confronting a cold spell, while the population as a whole suffered increased mortality. One must keep in mind that tracking by geolocator only provides data from recaptured, surviving individuals. Individuals differ in their response to abiotic stressors [36], and those not returning may have died due to an inappropriate response strategy. Because of spatial and temporal differences in climate change [1], long-distance migrants might be particularly challenged in their responses. For migratory birds the ability to combine external and internal stimuli appears to be essential for successful organization of the annual cycle. Understanding how species, populations, and even individuals respond to the changing climate and its associated weather extremes can help to predict the consequences for their population dynamics. Large phenotypic plasticity is likely to play a crucial role for population viability under the rapidly changing environment.

## Additional files

**Additional file 1.** Video of semi-collared flycatcher spring migration progression tracked by light-level geolocators in relation to temperature anomalies in 2014 and 2015.

**Additional file 2.** Raw sunrise and sunset data recorded by the geolocators.
